# Eosinophilic pneumonia associated with daptomycin: a case report and a review of the literature

**DOI:** 10.1186/1752-1947-5-13

**Published:** 2011-01-17

**Authors:** Andreas S Kalogeropoulos, Sotirios Tsiodras, Dionysios Loverdos, Panagiotis Fanourgiakis, Athanasios Skoutelis

**Affiliations:** 1 5th Department of Internal Medicine and Infectious Diseases, "EVANGELISMOS" General Hospital, 45-47 Ipsilantou Street, 106 76 Kolonaki, Athens, Greece; 2 4th Academic Department of Internal Medicine and Infectious Diseases, University of Athens Medical School, Attikon University Hospital, Athens, Greece

## Abstract

**Introduction:**

Although several studies did not demonstrate that daptomycin may cause significantly higher rates of pulmonary adverse effects when compared with vancomycin or penicillinase-resistant penicillins, there have been a few case reports of severe pulmonary complications associated with daptomycin administration.

**Case presentation:**

A rare case of eosinophilic pneumonia occurring 10 days after daptomycin administration in a 78-year-old Caucasian man with possible infectious endocarditis is described. He developed new onset fever, up to 38.5°C, with bilateral pulmonary crackles on physical examination and with no signs of severe respiratory failure. A chest computed tomography-scan showed bilateral nodular consolidations with air bronchograms and pleural effusions. Immediate discontinuation of daptomycin was followed by vigorous improvement of clinical signs and symptoms with progressive resolution of pulmonary consolidations a month later.

**Conclusion:**

Physicians should be aware of this rare but serious complication during daptomycin treatment, and prompt discontinuation of the offending agent, with or without additional supportive treatment, must occur immediately.

## Introduction

Eosinophilic pneumonia (EP) belongs to a heterogeneous group of lung diseases characterized by pulmonary infiltrates and increased numbers of eosinophils in lung tissue or broncho-alveolar lavage (BAL) fluid, with or without increased levels of eosinophils in the peripheral blood [1]. Acute EP due to drugs or toxins has similar clinical, radiographic and histopathologic manifestations to idiopathic acute or chronic EP, making the distinction of these entities difficult. The most common drugs associated with EP are antibiotics and anti-inflammatory drugs [2]. A complete and updated list of drugs suspected of causing lung disease can be found on a website maintained by the Groupe Etude de la Pathologie Pulmonaire Iatrogene at http://www.pneumotox.com.

Daptomycin, an antimicrobial agent of the cyclic lipopeptide group of antibiotics, has an outstanding coverage for Gram-positive bacteria and is licensed for the treatment of bacteraemia and right-sided endocarditis due to methicillin-susceptible and methicillin-resistant *Staphylococcus aureus *[3]. It is also effective for vancomycin-resistant *enterococci *[3]. Although daptomycin has a favorable adverse effect summary, and even though several retrospective studies did not show significantly increased incidence of pulmonary adverse drug reactions when compared to other anti-microbial agents [4-7], recently published case reports pointed out serious respiratory complications associated with daptomycin [8-11].

We present a case of pulmonary infiltrates and broncho-alveolar lavage eosinophilia occurring during treatment with daptomycin in a patient with possible infectious endocarditis (IE). In this particular case, and in contrast to previously published reports, our patient did not develop severe respiratory failure, and direct discontinuation of daptomycin without the systemic administration of corticosteroids was associated with the progressive and complete resolution of clinical manifestations and laboratory disturbances.

## Case presentation

A 78-year-old Caucasian man, with a history of coronary artery disease, presented with symptoms of acute congestive heart failure (CHF) including dyspnea at rest, orthopnea and paroxysmal nocturnal dyspnea.

The patient had a history of a transurethral prostatectomy (TURP) one month before admission. A week after the TURP, he developed a fever of 38.5°C that was considered a manifestation of a post-operative urinary tract infection and was treated empirically with oral ciprofloxacin 500 mg twice daily. The fever did not respond and treatment changed to oral amoxicillin/clavulanic 1 g twice daily and intramuscular netilmicin, 300 mg once daily. The fever resolved and no other clinical manifestations developed until the day of admission to our hospital. Regarding his past medical history, he was a non-smoker, he had no known allergies and he did not mention any recent travels.

**Figure 1:**
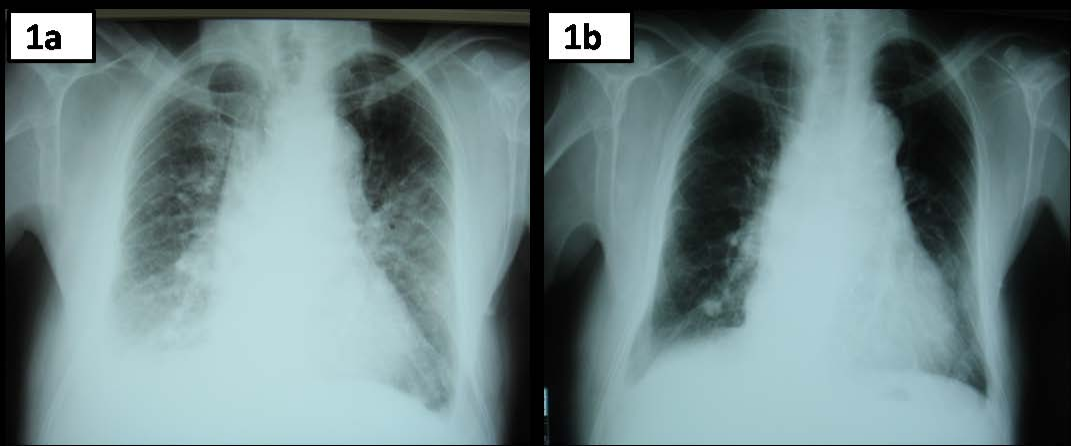
**Chest Radiograms.** a. Chest X-ray demonstrating bilateral perihilar alveolar edema with a "butterfly" appearance and bilateral pleural effusions. b. Chest X-ray after pharmaceutical treatment for the congestive heart failure symptoms. Most of the initially appeared radiographic findings have been almost completely resolved.

On admission, he was afebrile. His blood pressure was 120/55 mmHg, his heart rate 105/minute and his SaO_2 _was 92% on ambient air. The remaining physical examination revealed decreased breath sounds at both lung bases and inspiratory crackles at the lower pulmonary fields bilaterally, a 4/6 diastolic heart murmur at the lower left parasternal area and a 4/6 systolic heart murmur at the right upper parasternal area. Laboratory studies revealed a leukocyte count 8350/μL, hematocrit 36.8%, platelet count 270,000/μL and C-reactive protein (CRP) 1.0 mg/dL (normal range <0.5 mg/dL). A chest radiogram showed bilateral perihilar alveolar edema with a "butterfly" appearance and bilateral pleural effusions (Figure 1a). A transesophageal, two-dimensional Doppler echocardiogram showed a tricuspid aortic valve with a mobile vegetation of 9 mm in length on the right cusp and the presence of severe aortic valve regurgitation with possible perforation of the left cusp. Moderate mitral valve regurgitation was present as well, whereas the ejection fraction was 55%.

**Figure 2:**
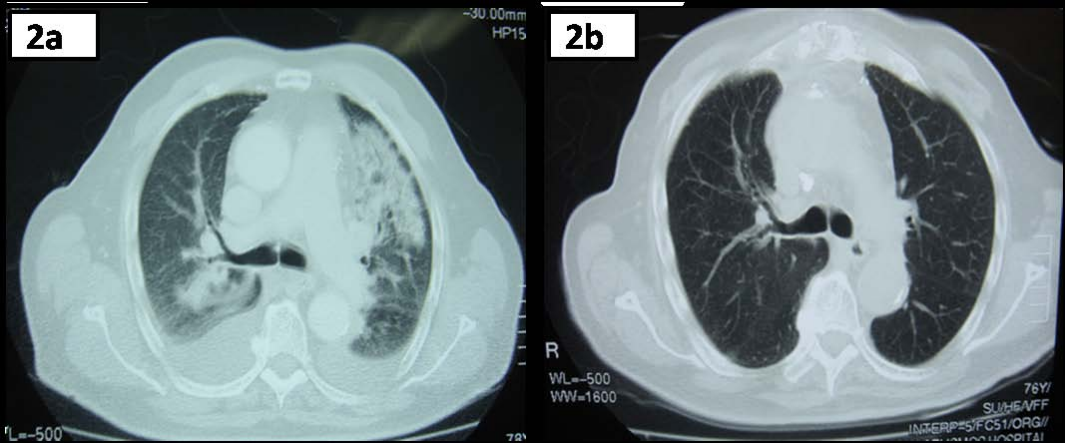
**Chest HRCT-scans.** a. Chest HRCT-scan demonstrating bilateral irregularly shaped nodular consolidations with air bronchograms and bilateral pleural effusions. b. Chest HRCT-scan, one month after daptomycin discontinuation, demonstrating complete resolve of nodular consolidations and bilateral effusions.

Following emergent treatment of CHF, all symptoms and physical signs were completely resolved. Additionally, a new chest radiogram showed significant improvement of the aforementioned radiographic findings (Figure 1b). Six sets of blood cultures from three separate body sites, drawn over 24 hours, were negative for a common bacterial pathogen. Considering the patient's previous history of TURP, and the previous admission of an antimicrobial regimen, empirical treatment for IE due to possible resistant enterococci was initiated including ampicillin 12 g daily, gentamicin 80 mg thrice daily and daptomycin (8 mg/kg) once daily. The patient responded positively to the empirical treatment until day 10, when he developed a new onset fever up to 38.5°C, accompanied by chills and diaphoresis. Physical examination revealed new onset crackles, predominantly at the left upper and medial pulmonary fields. The patient also showed significant hypoxemia with arterial blood gases analyses revealing a pH of 7.44, an oxygen saturation of 88%, a partial pressure of oxygen of 58 mmHg and a partial pressure of carbon dioxide of 38 mmHg, while breathing on ambient air. Laboratory studies revealed a leukocyte count of 9970/μL, with 78.3% neutrophils and 2.3% eosinophils. The erythrocyte sedimentation rate was 79 mm/h and CRP was 16.1 mg/dL. A chest x-ray was immediately performed demonstrating bilateral non-cavitating, reticulo-nodular infiltrates. All blood cultures were negative. The patient was treated with supplemental oxygen to maintain an oxygen saturation >92% and an additional empirical antimicrobial regimen for suspected health care acquired pneumonia (HCAP) was initiated (intravenous moxifloxacin 400 mg once daily and meropenem 3 g thrice daily). Inhaled corticosteroids and bronchodilators were also administrated. The high resolution chest computed tomography (chest HRCT) disclosed patchy areas of consolidation with ground-glass peripheral opacities and bilateral pleural effusions (Figure 2a). Urine examination for *S. pneumoniae *and *Legionella antigen *was negative. Serology for *Chlamydia pneumoniae, Mycoplasma pneumoniae*, *Bartonella spp, Coxiella burnetii*, *Brucella *and *Cytomegalovirus *was negative. The serological screening was negative for auto-immune markers (anti-nuclear antibodies, *cytoplasmic *and *perinuclear *anti-neutrophil cytoplasmic antibodies and anti- double-stranded DNA antibodies) as well. Despite the treatment, there was no clinical improvement. A thoracocentesis with a collection of pleuritic fluid for analysis was performed and the latter revealed a transudate with 6700 nucleated cells (70% lymphocytes, 15% eosinophils, 15% neutrophils). In addition, cultures for acid fast-bacilli and adenosine deaminase activity test of the pleuritic fluid were negative. To further investigate the nature of the aforementioned clinical syndrome a bronchoscopy with BAL was carried out, which disclosed 480 nucleated cells/μL (55% macrophages, 27.5% eosinophils, 12.2% neutrophils and 5.3% lymphocytes). Additional cultures for acid fast-bacilli, fungal and parasitic infections were also negative. Given the above findings the diagnosis of EP was made, daptomycin (as a probable cause of EP) was replaced by linezolid and moxifloxacin with meropenem were discontinued. Twenty-four hours after the daptomycin withdrawal the fever resolved completely. During the following seven days a significant improvement of the clinical and radiographic findings occurred, whereas CRP was within the normal range. One month later, a follow up chest-HRCT was normal (Figure 2b). The Naranjo causality scale yielded a score of 7 suggesting a probable adverse reaction due to daptomycin [12].

## Discussion

Eosinophilic pneumonia is a rationally uncommon entity and has been associated with several medications and chemicals, with antibiotics and non steroidal anti-inflammatory drugs being the most common eliciting factor [9]. The pathophysiology of EP is thought to involve the triggering of immune response due to an offending agent (for example, a drug or an infecting pathogen), principally expressed through antigen presentation by alveolar macrophages. This process may consequently provoke the recruitment of T-helper 2 (Th2) lymphocytes that sequentially release interleukin-5. Further eosinophil migration into the alveoli is facilitated through various mechanisms. Initially, interleukin-5 may promote significant eosinophil production and resettlement in the pulmonary alveoli. In addition, alveolar macrophages can excrete eotaxin, a cytokine that selectively recruits eosinophils by inducing their chemotaxis, which in turn may promote further eosinophil localization into the lungs [1].

Drug-induced EP can appear either as an acute or as a chronic syndrome that may occur within days or weeks after starting the offending agent. Diagnosis usually requires synthesis of information including clinical history, laboratory data and radiologic findings [13]. Patients with EP normally have cough and dyspnea for several days or weeks and may have a rash and/or fever. In acute patterns of EP patients may appear to have symptoms of severe dyspnea and hypoxemia resembling acute lung injury (PaO_2_/FiO_2 _<300 mmHg) or acute respiratory distress syndrome (PaO_2_/FiO_2 _<200 mmHg). It typically appears as areas of consolidation and ground-glass opacity on CT imaging, usually involving the peripheral pulmonary parenchyma. In addition, it may or may not be associated with peripheral blood eosinophilia, however pulmonary eosinophilic infiltrates or BAL eosinophilia are the corner stone for the diagnosis of EP [8]. A lung biopsy can verify the diagnosis but is not always a requisite given a typical clinical appearance and consistent laboratory and radiographic findings. In addition, according to the criteria of Solomon and Schwarz, the diagnosis of drug induced EP requires further evidence of pneumonitis with the aforementioned features, throughout treatment, with a drug that has the potential to provoke this syndrome. Infectious causes of eosinophilia, such as fungal or parasitic infections, need to be excluded, whereas clinical improvement should ensue drug cessation and symptoms should reappear after a rechallenge [2]. In our case, most of the criteria for the diagnosis of eosinophilic pneumonia were fulfilled. In particular, the patient developed fever and an abrupt abatement of respiratory function with hypoxemia during the treatment with an offending agent like daptomycin. However, the aforementioned syndrome did not progress to a severe respiratory failure and the patient did not require mechanical or non-mechanical ventilation. The arterial blood gases analysis revealed an acute lung injury with a PaO_2_/FiO_2 _ratio being 276. Moreover, in imaging studies with chest-HCRT he developed the characteristic pattern of bilateral peripheral consolidations and ground-glass opacities that we usually find in cases with eosinophilic pneumonia. Finally, BAL fluid examination revealed significant eosinophilia, a condition that is fundamental for the diagnosis of EP, whereas parasitic and fungal infections were excluded. However, before the accomplishment of the bronchoscopy procedure, we considered it essential to carry out a serological screening for autoimmune markers and a thoracocentesis, in order to examine the pleuritic fluid. Indeed, pleuritic fluid analysis revealed a transudate while cultures for acid fast bacilli and ADA test were negative; findings that were inconsistent with the possibility of tuberculosis as a cause of the clinical syndrome in our case. In addition, serological screening for autoimmune markers was also performed with the intention of excluding diseases of autoimmune origin, such as small vessel vasculitis, or systemic lupus erythematosus, conditions that may both provoke significant pulmonary lesions and non-infectious endocarditis [14,15].

Patients with idiopathic EP often require systemic corticosteroids treatment whereas those with drug induced EP demonstrate significant improvement, only with offending agent withdrawal. However, cases with persistent symptoms may need treatment with systemic corticosteroids and additional respiratory support with supplemental oxygen and assisted ventilation[16].

The EP described in our case is most likely attributable to an adverse drug reaction due to daptomycin administration. The patient had no history of chronic primary lung disease and he developed significant pulmonary abnormalities early after daptomycin initiation and had a remarkable improvement soon after daptomycin discontinuation. To the best of our knowledge, only five cases of EP associated with daptomycin have been reported thus far, but they should be considered in individuals who receive the drug and develop new pulmonary infiltrates [8-11]. In the majority of these reports (80%) [8,10,11] patients developed severe respiratory failure requiring systemic corticosteroids administration, intubation and assisted ventilation or supplemental oxygen and bimodal intermittent airway pressure support. In fact, in two of these cases persistent complete recovery did not occur and patients became chronically steroid dependent. Unfortunately, we are not able to make any comments regarding the association of the severity of the symptoms and the daptomycin dosage, since the daptomycin dosage regimen was not referred. In our patient, daptomycin was administered in high doses (8 mg/kg) in view of the fact that in previous studies higher doses of daptomycin were more effective and well tolerated when compared to other antimicrobial agents [17]. Additionally, our patient did not receive any systemic corticosteroid treatment since clinical presentation was not associated with severe respiratory failure and the patient exhibited significant clinical improvement after daptomycin discontinuation.

Daptomycin's toxicity mechanism remains uncertain and further *in vitro *and *in vivo *studies are necessary in order to elucidate its toxicity biochemical pathways. The primary mechanism of action involves calcium-dependent transitions, which are responsible for conformational changes of the daptomycin molecule that allow interactions with cytoplasmic membrane, enhancing daptomycin-cytoplasmic membrane binding capacity and cytoplasmic membrane permeability. The latter may induce significant leakage of intracellular ions, such as potassium. It has been recently demonstrated that synthetic surfactant binds to daptomycin and diminishes its antibacterial activity [18]. Therefore, we may assume that the administration of daptomycin for long periods of time could lead to increased accumulation of the drug near the alveolar epithelial surface, which subsequently may cause severe epithelial injury and organized pneumonia. Furthermore interaction of daptomycin with pulmonary surfactant may result in the deterioration of lipid integrity in the alveolar space, which in turn may trigger and conserve an inflammatory process [9].

## Conclusion

Daptomycin is a relatively new drug extensively used in tertiary health care units and in intensive care practice with excellent results regarding its antimicrobial activity. Although extremely rare, daptomycin-induced EP must be considered for patients who receive the drug and develop new unexplained pulmonary infiltrates. Significant morbidity and mortality may occur if this condition remains unrecognized and not properly treated in a timely fashion. Finally, further investigation through experimental and clinical studies needs to be completed in order to elucidate the exact mechanism behind this rare yet grave adverse drug reaction.

## Consent

Written informed consent was obtained from the patient for publication of this case report and any accompanying images. A copy of the written consent is available for review by the Editor-in-Chief of this journal.

## Competing interests

The authors declare that they have no competing interests.

## Authors' contributions

All authors are aware of and approved the manuscript being submitted to this journal. AK has made substantial contributions in drafting and revising the manuscript. ST, DL, PF and AS have been involved in revising the manuscript critically for important intellectual content. AS has given final approval of the version to be published.
